# The effectiveness of exercise therapy and education plus cognitive behavioral therapy, alone or in combination with total knee arthroplasty in patients with knee osteoarthritis – study protocol for the MultiKnee trial

**DOI:** 10.1186/s12891-021-04924-z

**Published:** 2021-12-20

**Authors:** Maren Falch Lindberg, Arild Aamodt, Mona Badawy, Ingvild B. Bergvad, Petter Borchgrevink, Ove Furnes, Caryl Gay, Stig Heir, Inger Holm, Kari Indrekvam, Nina Kise, Bjørn Lau, Jon Magnussen, Tor Kjetil Nerhus, Turid Rognsvåg, Daniil E. Rudsengen, Tone Rustøen, Søren T. Skou, Jan Stubberud, Milada S. Småstuen, Anners Lerdal

**Affiliations:** 1 Department of Surgery, Lovisenberg Diaconal Hospital, Pb 4970 Nydalen, 0440 Oslo, Norway; 2 Department of Nursing Science, Faculty of Medicine, University of Oslo, Pb 1072 Blindern, 0316 Oslo, Norway; 3 Coastal Hospital in Hagevik, Department of Orthopedic Surgery, Haukeland University Hospital, Bergen, Norway; 4 Institute of Health and Society, Faculty of Medicine, University of Oslo, PB 1072 Blindern, 0316 Oslo, Norway; 5 Department of Pain and Complex Disorders, St Olavs Hospital, Prinsesse Kristinas gate 3, 7030 Trondheim, Norway; 6 Norwegian University of Science and Technology, Høgskoleringen 1, 1491 Trondheim, Norway; 7 Department of Clinical Medicine, Faculty of Medicine, University of Bergen, Bergen, Norway; 8 Department of Orthopaedic Surgery, Haukeland University Hospital, Bergen, Norway; 9 Department of Family Health Care Nursing, University of California San Francisco, 505 Parnassus Ave, San Francisco, CA 94122 USA; 10 Martina Hansens Hospital, Dønskiveien 8, 1346 Gjettum, Norway; 11 Department of Acute Medicine, Oslo University Hospital, Pb 4956 Nydalen, 0424 Oslo, Norway; 12 Department of Psychology, Faculty of Medicine, University of Oslo, PB 1072 Blindern, 0316 Oslo, Norway; 13 Department of Sports Science and Clinical Biomechanics, University of Southern Denmark, 5230 Odense, Denmark; 14 Department of Physiotherapy and Occupational Therapy, Næstved, Slagelse and Ringsted Hospital, 4200 Slagelse, Denmark; 15 Institute of Clinical Medicine, Faculty of Medicine, University of Oslo, PB 1072 Blindern, 0316 Oslo, Norway

**Keywords:** Total knee arthroplasty, Osteoarthritis, Cognitive behavioral therapy, Exercise therapy

## Abstract

**Background:**

One in five patients report chronic pain following total knee arthroplasty (TKA) and are considered non-improvers. Psychological interventions such as cognitive behavioral therapy (CBT), combined with exercise therapy and education may contribute to reduced pain an improved function both for patients with OA or after TKA surgery, but the evidence for the effectiveness of such interventions is scarce. This randomized controlled trial with three arms will compare the clinical effectiveness of patient education and exercise therapy combined with internet-delivered CBT (iCBT), evaluated either as a non-surgical treatment choice or in combination with TKA, in comparison to usual treatment with TKA in patients with knee OA who are considered candidates for TKA surgery.

**Methods:**

The study, conducted in three orthopaedic centers in Norway will include 282 patients between ages 18 and 80, eligible for TKA. Patients will be randomized to receive the exercise therapy + iCBT, either alone or in combination with TKA, or to a control group who will undergo conventional TKA and usual care physiotherapy following surgery. The exercise therapy will include 24 one hour sessions over 12 weeks led by a physiotherapist. The iCBT program will be delivered in ten modules. The physiotherapists will receive theoretical and practical training to advise and mentor the patients during the iCBT program. The primary outcome will be change from baseline to 12 months on the pain sub-scale from the Knee Injury and Osteoarthritis Outcome Score (KOOS). Secondary outcomes include the remaining 4 sub-scales from the KOOS (symptoms, function in daily living, function in sports and recreation, and knee-related quality of life), EQ-5D-5L, the Pain Catastrophizing Scale, the 30-s sit-to-stand test, 40-m walking test and ActiGraph activity measures. A cost-utility analysis will be performed using QALYs derived from the EQ-5D-5L and registry data.

**Discussion:**

This is the first randomized controlled trial to investigate the effectiveness of exercise therapy and iCBT with or without TKA, to optimize outcomes for TKA patients. Findings from this trial will contribute to evidence-based personalized treatment recommendations for a large proportion of OA patients who currently lack an effective treatment option.

**Trial registration:**

Clinicaltrials.gov: NCT03771430. Registered: Dec 11, 2018.

## Background

Total knee arthroplasty is a common procedure to provide pain relief and improve function in patients with end-stage osteoarthritis (OA). In 2017, more than 750,000 TKA procedures were performed in the United States [1] and the number is expected to rise [2, 3]. A review of the literature [4] and our own empirical findings [5] indicate that 12–20% of TKA patients continue to experience moderate to severe pain 12 months after surgery and report dissatisfaction with their surgical outcome [6, 7]. There is currently no consensus on which pre- or postoperative intervention strategies or treatment options are effective for this subgroup of patients with a poor pain outcome. Moreover, as unfavorable outcome after TKA is poorly and arbitrarily defined across studies, countries and cultures, it is challenging to devise targeted interventions for subgroups of patients at risk for an inferior outcome trajectory.

Patients who develop chronic pain after TKA are characterized by a variety of physical and psychological health factors such as multiple painful sites [8], lower preoperative radiological degree of OA [9, 10], female gender, younger age [11], previous knee surgery [12], higher preoperative pain intensity [13], higher acute postoperative pain intensity [14] and poorer psychological state [8], catastrophic thinking [8] and fear of movement [15]. The extensive literature on risk factors suggests that to improve outcomes in TKA, both physical and psychological risk factors need to be addressed and optimized.

Exercise therapy and cognitive behavioral therapy (CBT) combined may significantly contribute to improving outcomes for patients following TKA, especially in patients at risk of poor pain outcome. Education and exercise therapy, and weight loss when relevant, are recommended as first-line treatments in patients with knee OA [16–18]. The effects of exercise are comparable to non-steroidal anti-inflammatory drugs and the results are sustained for up to 6 months [19]. In a previous randomized controlled trial (RCT), Skou and colleagues evaluated the effectiveness of TKA followed by a multimodal 12-week intervention, including exercise therapy and education, compared to the multimodal intervention alone in patients with moderate/severe OA scheduled for TKA [20]. While the patients who received TKA experienced greater improvements than those without surgery, both groups improved substantially over time, and only 26 and 32% of patients who received the multimodal intervention alone decided to undergo TKA during the 12- and 24-month follow-up period, respectively [20, 21].

CBT has shown promising results for OA patients in terms of reduced pain intensity [22, 23], improved function [22–24] and reduced health care costs [24]. Geng et al. also showed that perioperative psychotherapy, including CBT and medication, improved patient’s satisfaction 6 months after TKA among patients diagnosed with depression [25]. Despite these promising prior findings, no studies have tested the effectiveness of a combination of internet-delivered CBT (iCBT) and exercise therapy, either as a substitute for or as a supplement to TKA among patients with painful knee OA. While three recent studies found no effect for CBT on pain intensity in TKA patients with high levels of catastrophizing, these studies had small sample sizes [26, 27] and did not evaluate the effectiveness of CBT combined with physiotherapy [28]. This randomized controlled trial with three arms will compare the clinical effectiveness of patient education and exercise therapy combined with iCBT, either alone or combined with TKA, in comparison to routine treatment in patients with knee OA who undergo TKA surgery.

## Methods/design

### Study design

This is a multicenter, randomized trial of a 12-week exercise therapy and 10-week iCBT program delivered either alone or in addition to TKA, compared to TKA alone. Measurements will be taken at baseline and 3, 6, 12 and 24 months after the start of the intervention.

The protocol adheres to the SPIRIT guidelines [29] and the study was designed to conform with the CONSORT guidelines [30] for parallel-group randomized trials. The study was registered as a parallel-group RCT at ClinicalTrials.gov (NCT03771430) and was approved by the Regional Medical Research Ethics Committee of Health East of Norway (2017/968).

#### Participants

We will include 282 patients who meet the following inclusion criteria:

- Scheduled for primary TKA
- Age ≥ 18 and < 80
- ASA grade 1, 2 or 3
- Radiographic evidence of OA (Kellgren-Lawrence score 3 or 4)
- Body mass index < 40
- Able to read and write in Norwegian

Exclusion criteria:

- Previous unicompartmental or patellafemoral prosthesis in the index knee
- Large axis deviation or instability requiring use of hinged prosthesis
- Diagnosis of dementia
- Diagnosis of sero-postitive rheumatic disease

#### Procedure

**Fig. 1:**
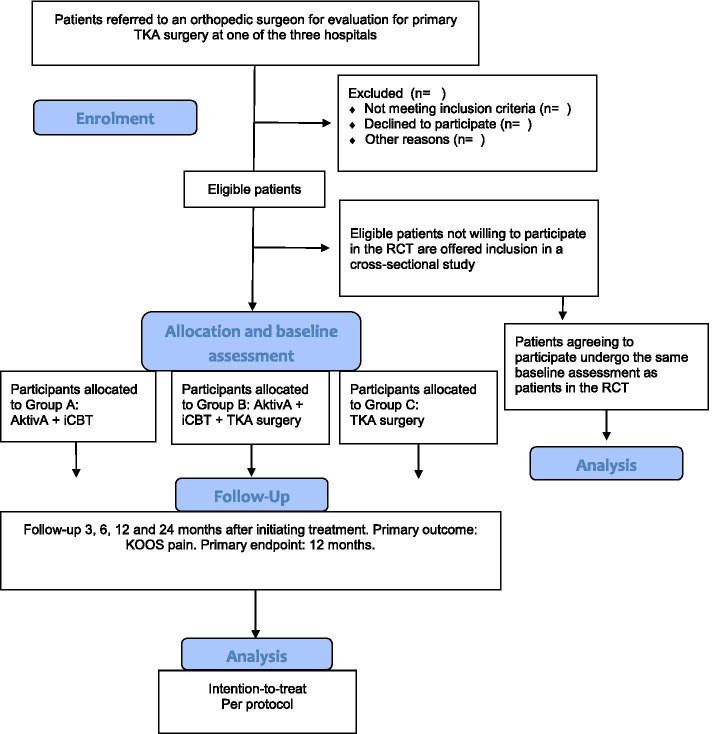
Flow chart. Patient flow through the study. Abbreviations: TKA, total knee arthroplasty; RCT, randomized controlled trial; AktivA, Active with Osteoarthritis; iCBT, internet-delivered cognitive behavioral therapy; KOOS, the Knee Injury and Osteoarthritis Outcome Score

People in need of evaluation for TKA are referred by their primary care physician to outpatient clinics at Lovisenberg Diaconal Hospital, Oslo, Coastal Hospital Hagevik, Bergen, or Martina Hansens Hospital, Bærum, all hospitals treat patients from all parts of Norway. These clinics specialize in performing TKA and are among the three hospitals in Norway with the largest volume of TKA procedures [31]. All patients are seen by an orthopedic surgeon who evaluates the patients’ symptomatology and function, as well as standard x-rays including anterior-posterior weight bearing view. The orthopedic surgeons will assess potential participants against the inclusion and exclusion criteria and then inform them verbally about the study and hand over an informational brochure. After the consultation, a research assistant will contact the patient and provide in-depth information about the study and invite them to participate. Eligible patients who are willing to participate will receive a link to an electronic consent form, which is signed using secure digital identification. A copy of the consent form is sent to their public digital mailbox account [32]. After signing the consent form and completing the baseline questionnaires, patients are randomized and receive information about their treatment assignment (A: Exercise therapy/iCBT non-surgical intervention group; B: TKA plus exercise therapy/iCBT intervention group; C: TKA-only control group). The physical tests are obtained again within 4 weeks prior to intervention start or surgery in order to standardize the timeline for baseline measurements, usually on the day before surgery or before their first session with physiotherapist. Figure 1 shows the follow-up measures obtained at 3, 6, 12 and 24 months after initiation of the intervention.

##### Randomization procedure, concealment and blinding

Patients who meet the eligibility criteria and are willing to participate will be randomized after signing the consent form (1:1:1 allocation ratio). The randomization schedule will be computer-generated by an independent statistician before initiating the trial using random permuted blocks of 3 or 6. The block randomization will be stratified by surgical center to account for variation in patient characteristics across the three centers. The randomization numbers will be concealed using sealed opaque C5 envelopes prepared by an independent staff member. The envelopes will be numbered sequentially and kept in a locked location and will only be accessible to the researchers involved in the recruitment of patients at each clinic. After the patient has signed the consent form, the next sequentially-numbered envelope placed in order will be opened by the research assistant, and the patient will be informed of the allocation.

##### Blinding

Blinding of participants and health personnel who deliver the intervention to the allocation groups will not be possible due to the nature of the intervention. The outcome assessors will not be involved in providing the intervention, and as the primary outcome is a self-reported measure, steps were not taken to blind them. The statistician performing the statistical analyses will be blinded to group allocation [33].

##### Cross-sectional study

Patients declining to participate in the RCT will be offered the option to participate in a separate cross-sectional study. The inclusion and exclusion criteria will be identical to the RCT. Patients who agree to participate in the cross-sectional study will complete the same baseline questionnaires as in the RCT and asked about the reasons they did not want to participate in the RCT.

### Interventions

#### Total knee arthroplasty

For participants allocated to one of the two surgical treatment groups (Group B and C), surgery will be performed within 4 weeks after obtaining the baseline measurements and no later than 8 weeks following the actigraph activity measures. If possible, the surgery will be performed by the same surgeon who assessed the patient at the outpatient clinic. Two different prosthesis designs (NexGen®, ZimmerBiomet, USA and Legion®, Smith&Nephew, USA) with different level of constraint will be used, based on the clinical situation and the preference of the surgeon. According to the hospital’s routine the patella will be not resurfaced, unless a posterior stabilized (PS) prosthesis is used. Patients are mobilized to standing the same day as surgery whenever possible and are allowed full weight bearing on the operated knee. Standardized physiotherapy with active and passive flexion and extension exercises is initiated on the day after surgery. Patients are mobilized on crutches and are usually discharged on day 2 following surgery. Within 2 weeks after discharge, patients in group B will commence on the MultiKnee program (described below) supervised by certified physiotherapists. Patients in group C will receive usual care physiotherapy in the municipalities typically consisting of exercise therapy with variable amount and quality of supervision, aiming at improving range of motion, strength, balance and gait.

#### The MultiKnee program

**Fig. 2:**
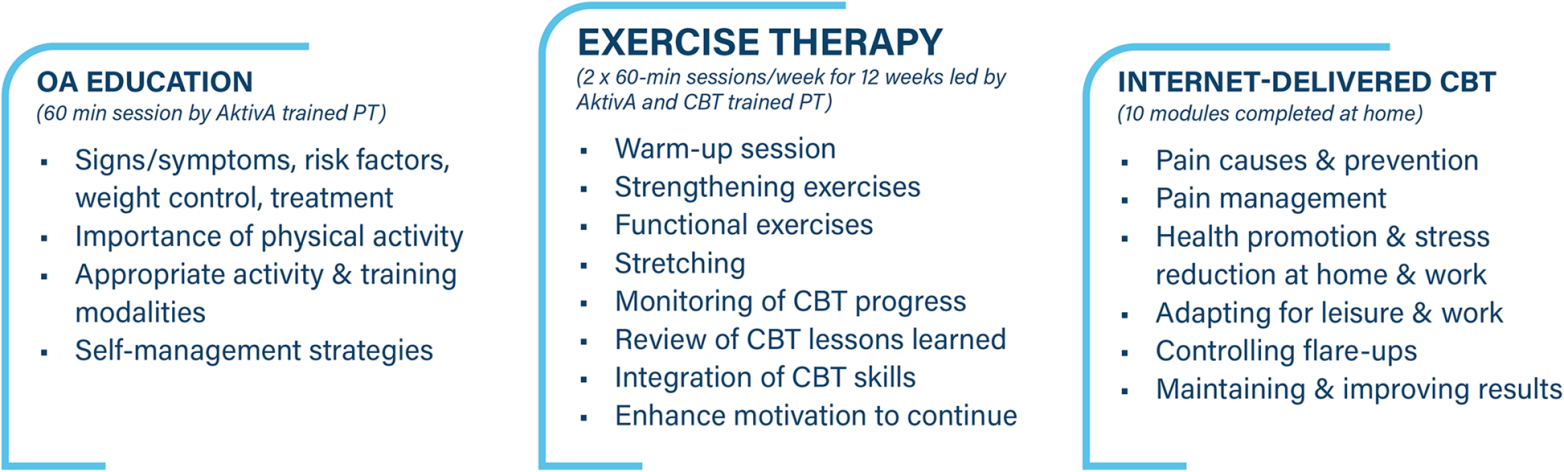
Overview of the MultiKnee program. Abbreviations: OA, osteoarthritis; CBT, cognitive behavioral therapy; AktivA, Active with Osteoarthritis; PT, physiotherapist

The program is reported according to the TIDieR checklist [34] and the CERT guidelines [35]. The MultiKnee program is a multidimensional intervention that consists of a combination of patient education, supervised exercise therapy delivered by a certified physiotherapist, and iCBT (Fig. 1). The participants allocated to group A can start their program immediately after randomization. Patients in group B will start the program within 2 weeks after discharge from hospital. The initial patient education will be delivered at each of the participating hospitals, whereas the exercise therapy will be delivered at a physiotherapy clinic near each patient’s home (Fig. 2).

#### Theoretical OA education

The theoretical patient education will be based on the education sessions from the evidence-based AktivA program [36], which is the Norwegian equivalent of Sweden’s Better Living with OsteoArthritis [37] and Denmark’s Good Life with osteoArthritis (GLA:D®) [38], developed specifically for hip and knee OA, and previously found to reduce pain and improve function and quality of life with sustained improvements for 12 months [38]. The aim of the program is to encourage the participant to engage in and take responsibility for the management of their OA. All hospitals will use the same presentation for the education sessions. The education session will last for 90 min and will focus on signs, symptoms and treatment of OA, the importance of exercise, lifestyle and self-management strategies. The education will be led by an AktivA trained physiotherapist and can be delivered in groups or individually. The physiotherapists will facilitate interaction and discussions during the session.

#### Exercise therapy

For the exercise therapy part of the intervention, the principles of the AktivA supervised exercise program will be applied. The AktivA program was developed based on the principles of the Osteoarthritis Research Society International (OARSI) treatment triangle [39], and has a bank of exercises that are suitable for patients with hip or knee OA. The aim of the program is to improve muscular strength, balance and functional stability. The duration of one exercise session is 45–60 min. Patients will exercise 2 times a week throughout the 12-week intervention. The exercise therapy program will consist of the following elements: warm-up, strengthening exercises, functional exercises, and stretching. The exercises will be performed on both legs, although the focus will be on the affected leg.

AktivA certified physiotherapists will deliver the exercise therapy and each participant will receive individual supervision. If the exercise therapy is delivered by a municipality-based physiotherapist who is not AktivA certified, one of the project physiotherapists will supervise the physiotherapist by assisting them to set up the exercise plan and monitoring by telephone every second week. The exercise therapy can be delivered individually or in groups. The patients will be monitored individually through 6 telephone mentor sessions by a trained physiotherapist to ensure that the program is tailored to each participant’s function and pain level and the program will be adjusted to the patient’s progression. Furthermore, if unacceptable pain or swelling occurs that is sustained the day after exercise, the intensity of the exercise will be reduced.

#### Internet-delivered CBT

The iCBT program is partially developed from relevant elements of a previous program developed by Braive Inc. [40] and was modified for OA patients by the MultiKnee team. The Braive platform provide internet-delivered courses for various mental health challenges. In the context of pain, CBT focuses on reducing pain and distress by modifying physical sensations, catastrophic and ruminative thinking, and maladaptive behaviors [41], in addition to enhancing self-efficacy [42, 43]. Based on these well-documented treatment principles, the iCBT program uses text and written exercises combined with animated videos. Three users have tested the prototype for the iCBT program and were interviewed about their experiences with the program, including feasibility, layout and relevance of the content. Their feedback was used to further refine the program. Furthermore, the program has been tested and further refined in a feasibility study with 15 patients, followed by a second round of testing by the same users.

The final program has been developed in two versions: Version A is for patients in group A who have not had a TKA and version B is for patients in group B who are receiving the program following TKA. Versions A and B are identical in content, except for the “persona” character who does not have TKA in version A but has had TKA surgery in version B. The iCBT program consists of 10 modules focused on training in several pain coping skills (i.e., pain mechanisms, relaxation techniques, changing unhelpful behavior, goalsetting, stress management, safety behavior, thinking errors, mindfulness, focused attention, postponing worries and rumination). Patients in groups A and B will receive access to the iCBT program at their first session with the physiotherapist and will then receive an individual user name and set a password. The program can be completed on a personal computer, tablet or smartphone. Patients will receive one new module each week for a total of 10 weeks. Patients complete the iCBT at home and at their own pace. CBT certified physiotherapists will support the patients with the iCBT program through 5 mentoring sessions by telephone. The aim with the mentoring sessions is to assess any barriers and motivate the patient to continue, as well as to help them integrate learned techniques into their exercise therapy sessions.

#### Physiotherapist training

Physiotherapists who deliver the MultiKnee program will receive training in two steps. First, they will participate in a full-day interdisciplinary certification course (AktivA) delivered by physiotherapists, an orthopedic surgeon, a nutritionist, and an experienced OA patient to provide a user perspective. Upon completion, the physiotherapists will be certified to deliver AktivA. The physiotherapists will also participate in a CBT education program led by a psychologist. The goal is to enable the physiotherapists to be mentors and motivators for the patients’ progress in the iCBT program, monitor the patients’ progression and help the patients to integrate their new skills into their exercise therapy program. The physiotherapists who mentor patients will receive a separate clinician access to the program, where they can monitor their patients’ progress. The clinician version of the program contains an electronic manual with a structured mentoring program including templates for each mentoring session related to the ten modules, advice on how to handle unexpected adverse effects of the program, and two learning modules with theoretical education about CBT for pain coping and Motivating Interview (MI) for conversation with the participants.

#### Crossover and discontinuation

Several precautions will be taken to reduce crossover or discontinuation. Immediately after randomization, the study assistants will telephone patients and inform them about their group assignment and what will happen during the study. Furthermore, physiotherapists will call patients every second week during the 12 weeks of the MultiKnee program, and have been trained to monitor and document adherence and encourage patients to stay in their assigned groups at least until the 12 week program has been completed. Patients who decide to cross over or discontinue participation will be contacted by the study assistants and asked about their reasons to crossover or discontinuation. If needed, they can be reassessed by an orthopedic surgeon. Patients who cross over to surgery will be asked to complete the Knee Injury and Osteoarthritis Outcome Score (KOOS) at the time of cross-over and to remain in the study for follow- up data collection to be included in the intention to treat analyses.

#### Protocol amendments

Following a feasibility study with 15 patients, but prior to inclusion of patients in the full-scale trial, several amendments were done. First, the inclusion criteria was changed from including patients at risk for an unsuccessfull outcome, to including all patients. The risk factors will still be assessed and analysed. Second, the sample size estimation was revised based on the new inclusion criteria and a third study site was added to our setup (Martina Hansens Hospital). Finally, major revisions were done for the iCBT program. The 15 patients in the feasibility study will be treated as a separate group and will not be included in the analyses for the full-scale MultiKnee trial.

#### Measurements

All measurements used in this study are shown in Table 1. Self-reported data will be collected electronically using the University of Oslo’s Service for Sensitive Data, a secure platform for collecting, storing, analyzing and sharing sensitive data in compliance with Norwegian privacy and research regulations [44].

**Table 1:** Study measures

**Construct assessed**	**Data collection instrument**	**Time of collection**
**Primary outcome measure**	*Patient-reported outcomes*	
Pain	Pain subscale of the KOOS	0, 3, 6, 12 and 24 months
**Secondary outcome measures**	**Data collection instrument**	**Time of collection**
*Patient-reported outcomes*		
Symptoms, ADL, QOL, sport & recreation	Four individual subscales of the KOOS	0, 3, 6, 12 and 24 months
Pain intensity, sites, & interference with functioning	Brief Pain Inventory	0, 3, 6, 12 and 24 months
Health-related quality of life	EuroQol-5D-5L	0, 3, 6, 12 and 24 months
Ability to forget about the knee	Forgotten Joint Score	0, 3, 6, 12 and 24 months
Pain catastrophizing	Pain Catastrophizing Scale	0, 3, 6, 12 and 24 months
Anchor measures of satisfaction	Patient acceptable symptom state	0, 3, 6, 12 and 24 months
	Treatment failure	3, 6, 12 and 24 months
	Global perceived effect	3, 6, 12 and 24 months
*Objective measures*		
Functional lower extremity test	30-s sit-to-stand test	0, 3, 6, 12 and 24 months
Adverse events	Treatment records, hospital records, questionnaire	Continuously – 12 monts
**Other measures**		**Time of collection**
*Patient-reported outcomes*		
Sleep quality	Pittsburgh Sleep Quality Index	0, 3, 6, 12 and 24 months
Mood states	Hospital Anxiety and Depression Scale	0, 3, 6, 12 and 24 months
Pain-related fear of movement	Fear-Avoidance Belief Questionnaire	0, 3, 6, 12 and 24 months
Health locus of control	Health Locus of Control Scale	0, 3, 12 and 24 months
Self-reported level of physical activity level and readiness for change	HUNT2, Stages of Change physical activity	0, 3, 6, 12 and 24 months
Digital health literacy	The eHealth Literacy Questionnaire	0 and 6 months
Health literacy	The International Health Literacy Population survey Questionnaire 2019–2021	0 and 6 months
Comorbidity	Self-Administered Comorbidity Questionnaire	0 months
*Objective measures*		
Time in active position/number of steps	ActiGraph Professional Single Axis accelerometer	0, 6, 12 and 24 months
Physical function -walking	40-m fast paced walk test	0, 3, 6, 12 and 24 months
Lower body strength and balance	Stair climb test	0, 3, 6, 12 and 24 months
Body mass index	Weight from baseline to follow-up	0, 3, 6, 12 and 24 months
Radiological assessments	Weightbearing AP, lateral view, Rosenberg view and long leg weightbearing AP view (HKA)	0 and 12 months
*Registry-based data*		
Use of health care resources	KUHR-system	0 to 24 months
	Norwegian Patient Registry	0 to 24 months
	FD trygd social security data base	0 to 24 months
	Norwegian Prescription Database	0 to 24 months
	The Norwegian Arthroplasty registry	0 to 24 months

Abbreviations: *ADL* Activities of daily living, *KOOS* Knee injury and Osteoarthritis Outcome Score

#### Primary outcome

The primary endpoint is change from baseline to 12-month follow-up on the KOOS pain subscale. The KOOS is a knee joint specific questionnaire with 42 items designed to assess patients’ experiences of problems with their knees during the past week. Higher scores indicate less pain. The KOOS has been validated for use in TKA and has been shown to be valid, reliable and responsive [45]. In addition to an intention-to treat analysis, the following sensitivity analyses will be performed: per protocol-analysis and as-treated analysis (described in detail in the section Statistical analysis). The KOOS has five subscales: Pain (9 items), Symptoms (5 items), ADL Function (17 items), Sport and Recreation Function (5 items), Quality of Life (5 items). The five dimensions are scored separately. All items are scored on a Likert scale with five categories scored from 0 (no problems) to 4 (extreme problems). Each subscale score is calculated as the sum of the included items, and transformed to a 0–100 scale, with 0 representing extreme problems and 100 representing no knee problems [45].

#### Secondary outcomes

The remaining KOOS subscale scores (Symptoms, ADL, Sports and Recreation, Quality of Life) will be secondary outcomes.

Functional lower extremity strength will be measured using the 30-s sit-to-stand test [46]. This test is performed using a chair of standard height without arms. The participant is encouraged to complete as many full stands as possible within 30 s. In a systematic review [47], this instrument is a recommended sit-to-stand measure for patients with knee OA.

Pain intensity and interference with functioning will be measured using the Brief Pain Inventory (BPI) [48]. The BPI consists of four items that measure pain intensity (on an 11-point numeric rating scale from 0 to 10), one item on pain relief, seven items on pain interference with functioning, and a body map to localize the pain. The BPI was a reliable and valid measure of pain in a Norwegian sample [49].

Health-related quality of life will be measured with the widely-used EuroQol EQ-5D-5L, which has five items assessing different dimensions of health status (mobility, self-care, usual activities, pain/discomfort, anxiety/depression). The five dimensions can be used to calculate a weighted health state index score ranging from less than 0 (where 0 is the value of a health state equivalent to dead, negative values representing values as worse than dead) to 1 (the value of full health). According to the literature, a clinically relevant difference in the EQ-5D index between the groups would be 0.08 [50]. An additional visual analog item assesses the respondent’s perception of his/her overall health. The EQ-5D-5L is simple to use, valid, responsive to change and reliable for group comparisons [51]. The EQ-5D-5L will be used to calculate quality-adjusted life years (QALYs) as a measure of benefit in cost-utility analyses.

The Forgotten Joint Score [52] will be used to measure the patients‘ability to forget about a joint as a result of successful total knee arthroplasty. Patients rate their agreement with 12 statements that range from 1 (never) to 5 (mostly). The raw score is transformed to a 0–100 score and then reversed to obtain the final score. A higher score indicates less awareness of the knee after TKA.

Pain Catastrophizing will be measured using The Pain Catastrophizing Scale (PCS) [53]. It consists of 13 items that assess three dimensions of catastrophizing (i.e., rumination, magnification, helplessness). The Norwegian version of the PCS has acceptable validity and reliability [54].

Patient-acceptable symptom state (PASS) and perceived treatment failure: will be assessed by the following single item question, used in similar trials [55, 56]. “When you think of your knee function, will you consider your current condition as satisfying? By *knee function*, you should take into account your activities of daily living, sport and recreational activities, your pain and other symptoms and your quality of life’. Answered by “yes” or “no”. Patients who indicated that their knee function is not satisfactory by answering “no” on the PASS question will be asked to complete a single item question related to treatment failure [57]: “Would you consider your current state as being so unsatisfactory that you think the treatment has failed?” answered by “yes” or “no”.

Global perceived treatment effect: Patients rate their level of knee problems compared to their condition before the intervention by choosing one of seven statements that describe their level of improvement/worsening [58, 59]. The statements range from “better – an important improvement” – to “worse – an important worsening”.

Adverse events (AE) and serious adverse events (SAE) will be recorded during the entire study period using medical records, physiotherapist-reported adverse events, and patient-reported adverse events using questionnaires that include open probe questioning to ensure that all AEs are recorded. Medical records will be checked at the primary endpoint (12 months) for all AEs and SAEs from inclusion until the 12-month follow-up, and will be assessed for severity by an adjudication committee independent of whether there is a causal relationship with the study treatment. An AE is defined as any undesirable experience during follow-up that leads to contact with the health care system, whereas an SAE is defined as any event that leads to hospitalization, prolonged in-hospital care or additional surgery, is life-threatening or results in permanent disability or damage, or death [60]. While crossover to surgery will not be considered an adverse event, it will be registered and will be important when evaluating the results of the trial.

#### Other measures

Sleep quality: Sleep disturbance in the past month will be measured with the Pittsburgh Sleep Quality Index [61]. It has good validity and reliability [62, 63].

Mood states (depression and anxiety) will be measured with the Hospital Anxiety and Depression Scale [64]. The scale consists of 14 items, seven on the depression subscale and seven on the anxiety subscale. The Norwegian version has excellent psychometric properties [65].

Pain-related fear of movement will be measured using the Fear-Avoidance Belief Questionnaire (FABQ) [66]. It consists of two subscales, fear-avoidance beliefs for work and physical activity, but only the subscale for physical activity will be used in this study. The Norwegian version of the FABQ has satisfactory validity [67].

The Health Locus of Control Scale will be used to measure patients’ beliefs about whether their health is controlled by internal or external factors [68]. The scale consists of 18 statements that form 3 subscales assessing patients’ health locus of control: Internal, Powerful Others, and Chance. Patients rate their agreement on a six-point Likert scale ranging from “disagree completely” to “agree completely”. Higher scores on a given subscale indicate stronger beliefs that the patients’ health is controlled by the focus of that subscale (i.e., internal factors, powerful others, or chance).

Self-reported physical activity levels and readiness for change in physical activity will be measured using the Stages of Change (SoC) [69] and the Hunt 2 for physical activity [70]. With the Hunt, patients rate the frequency of light and hard physical activity for a typical week during the past month. With SoC, patients state their readiness for physical activity from five stages: precontemplation, contemplation, preparation, action and maintenance [71].

The eHealth Literacy Questionnaire (e-HLQ) [72] will be used to measure patients level of digital health literacy prior to, and 6 months following surgery. The original e-HLQ consist of 35 items and 7 domains. In this study, 4 domains will be assessed: 1) using technology to process health information, 2) understanding of health concepts and language, 3) ability to actively engage with digital services, 4) motivated to engage with digital services. The scores range 1–4, with high scores indicating high e-health literacy [72].

The International Health Literacy Population survey Questionnaire 2019–2021(HLS19-Q47) [73] will be used to measure patients’ level of health literacy prior to, and 6 months following surgery. The original HLS-19-Q47 consist of 47 items and contains 4 domains. In this study, two domains will be assessed: 1) Health promotion, 2) General health literacy.

Comorbidity will be measured prior to randomization using the Self-Administered Comorbidity Questionnaire [74]. The question “Do you have any of the following problems?” is followed by 17 diseases plus an “other” category. Each question is followed by the questions “Do you receive treatment for it?” and “Does it limit your activities?”

#### Other measures - clinical assessments

Activity measure: The ActiGraph GT3X-BT Activity monitor [75, 76], a body-worn sensor system to capture and record physical activity, will be used to measure time in sedentary and active positions, the duration of activity, the number of steps during walking, and sleep/wake information. Patients will wear the ActiGraph for 1 week before treatment start and at 6, 12 and 24 months follow-up.

Performance-based tests include the minimum core set of measurements used to assess functional performance in people diagnosed with knee OA, as recommended by the Osteoarthritis Research Society International (OARSI) [47]:

The 40-m Fast-paced Walk Test will be used to measure physical function. The patient is instructed to walk as fast as possible for 40 m [47].

The Stair Climb Test will be used to measure patients’ lower body strength and balance by assessing the time in seconds in takes them to ascend and descend a flight of stairs [47].

Body mass index will be measured using the following algorithm: weight in kilograms/height in meters^2^ [77]. A healthy BMI range is between 18.5–24.9. BMI > 25 will be defined as overweight.

X-rays will be obtained to assess radiographic evidence of OA. Views will include weight-bearing AP, lateral, Rosenberg and long leg weight-bearing AP (HKA). OA severity grading will be performed according to the Kellgren-Lawrence grading system [78] and cartilage thickness.

#### Other measures – compliance, other treatments and registry-based data

Use of primary health care services will be measured using registry data from the KUHR-system (i.e., control and payment of reimbursements to health service providers), the Norwegian Patient Registry (NPR), the Norwegian Prescription Database and FD Trygd social security data base. All data retrieved from the registries will be anonymized and data from several sources will be linked to each patient using a unique ID number. Data on specialist health care services (i.e., revision surgery and deep prosthetic infections) will be collected from the Norwegian Arthroplasty registry [31] and medical records.

##### Compliance and other treatments

Patients will be asked to report any additional treatments related to their knee problems that they have used during the follow-up period using a questionnaire.

For patients in the intervention groups A and B, adherence to the exercise therapy program and the iCBT program will be registered by the physiotherapists at each telephone session throughout the 12 weeks as well as their progression and any adjustments using a structured log. We define poor compliance as completing less than 75% of the exercise therapy sessions, or less than 75% of the iCBT modules.

#### Power calculation and statistical analyses

The minimal perceptible clinical improvement for the KOOS has been determined to be 10 points [45], which is considered a minimal clinically important change (MIC). Statistical power will be set to 90%, the level of significance to 1% due to multiple testing, and the common standard deviation of change in the three groups is set to 16, based on a previous study from our group [79]. Thus, a sample size of 78 patients in each treatment group is required. Allowing for 20% drop-out, we will include 282 patients (i.e, 94 patients in each treatment group).

##### Statistical analyses

Data analyses will be performed using SPSS version 24.0 (IBM, Armonk, NY) and Stata version 16 [80]. For continuous outcomes, differences over time, including measures at baseline, 3, 6 and 12 months, will be analyzed using mixed models for repeated measures, with patients as random effects, and follow-up visits and treatment groups as fixed effects, while controlling for any baseline differences and randomization stratification factors (i.e., hospital). A 95% Confidence Interval excluding 10 points or more in the KOOS pain score will be interpreted as a lack of clinical meaningful difference. No imputation will be performed.

For categorical outcomes, appropriate non-parametrical tests will be used (e.g., Kruskal-Wallis test, Chi-square testing). *P*-values < 0.01 will be considered statistically significant, and 95% confidence intervals will be reported for all point estimates. Effect sizes will be calculated for group differences using Cohen’s coefficient *d*. A *d*-value ≥0.40 will be considered a clinically meaningful difference [81]. The occurrence of adverse events will be compared between groups at the 12 month follow-up using a poission regression model with a robust error variance.

The analyses at 24 months will follow a similar analysis strategy as described for the 12 months outcome.

##### Study-specific responder analysis

To guide clinical interpretation of the results, we will calculate study specific and subscale specific cutoff scores by subtracting the mean KOOS pain subscore for those reporting to have “unchanged” pain from those reporting “less pain” at 12 months on the 7 point global perceived effect scale ranging from “better – an important improvement” – to “worse – an important worsening” [82].

##### Cost-utility analysis

A societal perspective will be used, as recommended by Russel et al. [83]. A Markov decision model will form the theoretical framework for a cost-effectiveness analysis to estimate the costs and benefits for patients in the two intervention groups and the control group. The main variables will be QALYs based on the EQ-5D, combined with the use of health care resources including use of medication. The incremental cost-effectiveness ratio (ICER) will be used to summarize the cost-utility of each of the intervention groups, compared to the control group. Sensitivity analyses will be performed to test the stability of the conclusions.

##### Sensitivity analyses

The intention to treat analyses and the patient safety analysis will include all patients who were randomized. Furthermore, for the primary outcome, per-protocol analyses and as-treated analyses will be performed. For the per-protocol analysis, we will exclude patients who crossed over from non-surgical treatment to surgery, those who had low compliance with the intervention defined as completing < 75% of each of the elements of the intervention, and those in either of the surgical groups who did not undergo surgery. The as-treated analysis is expected to have four groups, the three original randomization groups as well as a group with those from group A crossing over to surgery.

#### Ethical perspectives

The Regional Medical Research Ethics Committee of Health East of Norway approved the study (2017/968). The Data Protection Officers at Lovisenberg Diaconal Hospital, Coastal Hospital Hagevik and Martina Hansens Hospital have evaluated and recommended the study. We will obtain informed written consent from all participants. Only the research group will have access to the data. We will depersonalize the data using a code number before statistical analysis. Participating in the intervention will require time and effort by the patients. The non-surgical treatment will be in line with current recommendations for knee OA, thus we do not anticipate any increased physical risks for the participants beyond usual care. Patients who are randomized to surgery will be informed about expected risks and benefits of surgery, according to the hospitals’ standard procedures. Patients randomized to non-surgical treatment can be reassessed by an orthopedic surgeon at any time during follow-up. If the patient and surgeon agree, the patient will be offered TKA. Patients who wish to crossover or discontinue their participation will be offered TKA operation if still needed.

## Discussion

This study will be the first to provide high-quality evidence of the effectiveness of an integrated intervention with patient education, physical exercise and CBT, delivered alone or in combination with TKA, on pain and functional outcomes in patients with knee OA. Findings from this trial will contribute to the development of evidence-based personalized treatment recommendations for a large proportion of OA patients.

If TKA surgery is found to be more effective when delivered in combination with non-surgical treatment, it may be introduced as a supplement to TKA to improve outcomes, in particular for patients who are at higher risk for a poor outcome if they undergo TKA. The non-surgical treatment may also be applicable as a standard first-line treatment option for patients eligible for TKA.

A unique characteristic of the study is that our non-surgical treatment aims to address and modify both psychological and physical factors. There is broad agreement that education, exercise therapy, and weight loss when relevant, are effective and should be the first-line treatment in OA [16–18]. Growing evidence suggest that psychological factors are associated with a poorer outcome following TKA, possibly due to lower adherence to non-surgical treatment. In a systematic review [84], patients with low self-efficacy, depression, anxiety, poor social support, and increased pain levels during exercise had poor adherence with exercise therapy. Furthermore, psychological factors and physical comorbidities can negatively influence each other in a bi-directional relationship [85, 86]. An intervention to reduce pain in OA patients should therefore use a biopsychological approach addressing physical and psychological impairments simultaneously. Turk et al. developed a cognitive-behavioral approach to pain management [87]. This method addresses several psychological factors that may impact pain intensity and disability, such as catastrophic thinking [88], fear-avoidance [89], low self-efficacy, helplessness and lack of perceived control [42, 43, 90, 91], as well as passive pain coping strategies [92]. Of these, pain-related catastrophic thinking and pain-related fear have particularly strong associations with both pain intensity and disability in patients with musculoskeletal pain [93] and knee OA [94]. Depression, pain catastrophizing and pain-related fear of movement are also prognostic factors for the transition from acute to chronic pain [93, 95].

Considering the limited health resources available, the health-economic aspects and evaluation of cost-utility included as a part of this study will be of particular importance. Among the 7161 patients who underwent primary TKA in Norway in 2019 [31], it is estimated that 10 to 34% experienced little to no benefit, or even worsening of their pain, suggesting use of health resources with questionable effect form a relatively large proportion. The use of resources is even higher when health care services following TKA are included. If successful, findings from this study may result in more personalized treatment options and more effective use of health care resources.

Our study has several strengths. It will be performed by a multidisciplinary research group, with a unique blend of professions (orthopedic surgeons, physiotherapists, pain specialists, orthopedic nurses, health economists and psychologists) stemming from strong competence environments (largest TKA centers in Norway: Lovisenberg, Oslo, Coastal Hospital Hagevik, Bergen, Martina Hansens Hospital, Bærum], the Norwegian Arthroplasty Register in Bergen, and the Pain Competence Center, St Olavs Hospital, Trondheim. The physiotherapists who deliver the intervention will be trained in both AktivA and CBT. Another major strength is that the iCBT part of the intervention has been developed in cooperation with users who have made significant contributions to its content. The study measurement instruments are valid and reliable, and a combination of self-reported measures and objective measures will be used.

There are some potential threats and limitations. Low or delayed enrollment may be a threat to this study. This point has been addressed by interviewing 10 patients about their impression of the study, willingness and barriers to participate. In an RCT on the effectiveness of TKA in addition to non-surgical treatment including exercise therapy, patients’ willingness to participate was high, even in the group that was randomized to non-surgical treatment alone (20). A lack of blinding may pose a threat. All follow-up examinations will be done by trained outcome assessors not involved in the delivery of the intervention. Furthermore, we will not be able to differentiate between the effectiveness of the patient education, exercise therapy and the iCBT, as all patients randomized to the two intervention groups will receive all of these interventions. Patients assigned to the exercise therapy and iCBT only group may decide to have surgery within the study period. They will, however, be encouraged to postpone surgery and cross-over will be recorded. Poor adherence to the intervention among patients and fidelity to the intervention protocol among physiotherapists are other potential threats. The physiotherapists will monitor patient adherence and discuss barriers to motivate the patient to continue. A psychologist will monitor the physiotherapists’ fidelity to the protocol when delivering the iCBT intervention and provide guidance and assistance as needed.

This randomized controlled study will provide high-quality evidence on the effectiveness of exercise therapy and iCBT, either as a separate treatment choice or combined with TKA, in comparison to TKA alone, in patients with knee OA who are considered candidates for TKA. The results may be of critical importance to develop individually tailored treatment options, and to improve the results for the 20% of patients who currently have questionable benefit from TKA.

## Data Availability

The dataset will be stored on the University of Oslo’s Service for Sensitive Data, in accordance with Norwegian ethical and legal requirements. Only selected researchers in the MultiKnee team will have access to the data. Upon completion of the trial, any requests for an anonymized minimal data set can be sent to the corresponding author. Final approval from the Data Protection Officer and the Regional Committees for Medical and Health Research Ethics will be required prior to release of an anonymized minimal data set. All results will be published in Open Access journals, in line with requirements from the Norwegian Research Councils Plan S policy for open publication [96].

## Abbreviations

AE: Adverse event
BPI: Brief Pain Inventory
FABQ: Fear Avoidance Belief Questionnaire
GLA:D: Good Living with Osteoarthritis
iCBT: Internet delivered cognitive behavioral therapy
KOOS: Knee Injury and Osteoarthritis Outcome Score
OA: Osteoarthritis
OARSI: Osteoarthritis Research Society International
PASS: Patient acceptable symptom state
PCS: Pain Catastrophizing scale
RCT: Randomized controlled trial
SAE: Serious adverse event
TKA: Total knee arthroplasty
